# Association between gene mutations and outcomes in Japanese high-risk AML patients: a phase 1/2 study of NS-87/CPX-351

**DOI:** 10.1007/s12185-025-03956-8

**Published:** 2025-02-27

**Authors:** Hideki Makishima, Taisuke Mikasa, Kento Isogaya, Toshihiro Miyamoto, Takuji Yamauchi, Akira Yokota, Masahiro Onozawa, Kiyoshi Ando, Yoshiaki Ogawa, Kensuke Usuki, Takahiro Yamauchi, Shuichi Ota, Satoru Takada, Yasuyoshi Morita, Takayuki Ishikawa, Katsuto Takenaka, Junya Kuroda, Naohiro Sekiguchi, Toshiro Kawakita, Yasushi Miyazaki

**Affiliations:** 1 Department of Hematology and Medical Oncology, Shinshu University School of Medicine, 3-1-1 Asahi, Matsumoto, 390-8621 Japan; 2 Clinical Development Department, Nippon Shinyaku Co., Ltd, Kyoto, Japan; 3 Data Science Department, Nippon Shinyaku Co., Ltd, Kyoto, Japan; 4 Department of Hematology, Kanazawa University, Kanazawa, Japan; 5 Department of Medicine and Biosystemic Science, Kyushu University Graduate School of Medical Sciences, Fukuoka, Japan; 6 Department of Hematology, Chiba Aoba Municipal Hospital, Chiba, Japan; 7 Department of Hematology, Hokkaido University Hospital, Sapporo, Japan; 8 Department of Hematology and Oncology, Tokai University School of Medicine, Isehara, Japan; 9 Forth Department of Internal Medicine, Mizonokuchi Hospital, Teikyo University School of Medicine, Kawasaki, Japan; 10 Department of Hematology and Oncology, University of Fukui, Fukui, Japan; 11 Department of Hematology, Sapporo Hokuyu Hospital, Sapporo, Japan; 12 Leukemia Research Center, Saiseikai Maebashi Hospital, Maebashi, Japan; 13 Department of Hematology and Rheumatology, Kindai University Faculty of Medicine, Sayama, Japan; 14 Department of Hematology, Kobe City Medical Center General Hospital, Kobe, Japan; 15 Department of Hematology, Clinical Immunology and Infectious Disease, Ehime University Graduate School of Medicine, Toon, Japan; 16 Division of Hematology and Oncology, Department of Medicine, Kyoto Prefectural University of Medicine, Kyoto, Japan; 17 Department of Hematology, National Hospital Organization Disaster Medical Center, Tachikawa, Japan; 18 Department of Hematology, NHO Kumamoto Medical Center, Kumamoto, Japan; 19 Department of Hematology, Atomic Bomb Disease Institute, Nagasaki University, Nagasaki, Japan

**Keywords:** Acute myeloid leukemia, CPX-351, *TP53* mutations, NGS

## Abstract

This phase 1/2 study investigated the association between genetic characteristics and outcomes for NS-87/CPX-351 in Japanese patients with high-risk acute myeloid leukemia. Blood samples collected from 29 patients were analyzed using a 70-gene next-generation sequencing panel. The most frequently mutated genes were *TP53* (44.8%), *TET2* (24.1%), *DNMT3A* (13.8%), and *NRAS* (13.8%). The rates of composite complete remission (CRc; complete remission [CR] or CR with incomplete hematologic recovery [CRi]) were comparable between patients with and without mutations in *TP53, TET2*, *DNMT3A*, and *NRAS* (*P* = 0.571 for all). Notably, patients with *TP53* mutations had a similar CRc rate (69.2% vs. 56.3%), but shorter overall survival (OS), event-free survival (EFS), and relapse-free survival (RFS) compared to patients with wild-type *TP53* (median OS: 7.43 vs. 18.18 months; *P* = 0.108, median EFS: 2.43 vs. 6.28 months; *P* = 0.012, median RFS: 1.48 vs. 10.19 months; *P* = 0.012). In conclusion, no gene mutations directly associated with the efficacy of NS-87/CPX-351 were found. While NS-87/CPX-351 achieved remission even in patients with *TP53* mutations, relapse risk was higher in these patients. Therefore, it is advisable to consider treatment strategies such as early transplantation after achieving remission with NS-87/CPX-351, especially in patients with *TP53* mutations.

**Supplementary Information:**

The online version contains supplementary material available at 10.1007/s12185-025-03956-8.

## Introduction

Acute myeloid leukemia (AML) is a disease characterized by clonal proliferation of immature myeloid cells and is associated with various cytogenetic abnormalities and gene mutations [1, 2]. Although most cases arise de novo, AML can also occur as a secondary malignancy that has antecedent hematologic disorders or that emerges after prior cytotoxic chemotherapy and/or radiation therapy for a neoplastic or non-neoplastic disease [3]. AML following a previous myeloid malignancy (e.g., myelodysplastic syndromes (MDS) and myeloproliferative neoplasms) or therapy-related AML is more common in elderly patients and tends to involve more mutated genes than de novo AML [4–6]. Specifically, patients with AML evolved from MDS are reported to have a high prevalence of mutations in *SRSF2*, *SF3B1*, *U2AF1*, *ZRSR2*, *ASXL1*, *EZH2*, *BCOR*, or *STAG2*, and patients with therapy-related AML are frequently found to have *TP53* mutations [4, 7–9]. Secondary AML, such as AML evolved from MDS or therapy-related AML, is an aggressive malignancy with a poor response to therapy and inferior overall survival (OS) due to the common presence of adverse-risk cytogenetic features [5, 6, 10–12]. Moreover, the genomic landscape differs between younger and older patients with AML. The genes *DNMT3A*, *TET2*, and *ASXL1*, which are associated with clonal hematopoiesis, are commonly mutated in the elderly, irrespective of clinical or preclinical state [13].

NS-87/CPX-351 is a liposomal encapsulation of cytarabine and daunorubicin at a fixed molar ratio of 5:1 [14, 15]. In a randomized phase 3 study (301 study) of patients aged 60 to 75 years with high-risk AML, including newly diagnosed therapy-related AML or AML with myelodysplasia-related changes (AML-MRC), NS-87/CPX-351 significantly improved median OS versus conventional 7 + 3 chemotherapy (9.56 months vs. 5.95 months; hazard ratio (HR) = 0.69 (95% confidence interval (CI) 0.52–0.90)) [16]. NS-87/CPX-351 was approved for the treatment of newly diagnosed therapy-related AML or AML-MRC in the US as well as in the EU. In Japan, a phase 1/2 study (NS87-P1-2 study) evaluated the pharmacokinetics, safety, and efficacy of NS-87/CPX-351 in elderly Japanese patients with high-risk AML. This study noted no apparent differences in efficacy and safety compared to the 301 study, and NS-87/CPX-351 was approved for the treatment of this high-risk population in Japan [17].

The association between gene mutations and outcomes for NS-87/CPX-351 has been evaluated in the 301 study and several real-world studies. However, the effect of gene mutation status on efficacy varies across studies. *TP53* mutations, which are commonly observed in high-risk AML patients, are an example. The 301 study found that patients with *TP53* mutations had a lower composite complete remission (CRc; complete remission [CR] or CR with incomplete hematologic recovery [CRi]) compared to the entire study population [16, 18]. In contrast, retrospective Italian and German studies found that CRc rate was similarly high in patients with and without *TP53* mutations, indicating differences in CRc rates between studies [19, 20]. Given the absence of a reported association between genetic characteristics and treatment outcomes for NS-87/CPX-351 in Japanese patients, we evaluated the genetic characteristics of elderly Japanese patients enrolled in the NS87-P1-2 study. A point to note is that the analysis in this study was performed as a post hoc test.

Our aim was to examine the potential association between genetic characteristics and outcomes for NS-87/CPX-351 in elderly Japanese patients with high-risk AML in order to explore gene mutations associated with the efficacy of NS-87/CPX-351.

## Materials and methods

### Study design

The full study design, patient eligibility criteria, and efficacy end points of the NS87-P1-2 study are described in detail elsewhere (Supplementary Material) [17]. AML was diagnosed by investigators based on the WHO 2017 criteria, and patients for whom any of the following were confirmed were eligible for the study: therapy-related AML, AML with a history of MDS, de novo AML with karyotypic abnormalities characteristic of MDS, or AML with a history of chronic myelomonocytic leukemia (CMML). We analyzed blood samples collected from 29 patients at diagnosis prior to treatment; patients had given written informed consent for exploratory genetic analyses in advance. All patients were followed up for two years after the last patient received NS-87/CPX-351. The study completion date was October 26, 2023. This study was approved by the institutional review board of each participating hospital in accordance with the Declaration of Helsinki.

### Genetic analysis

DNA was extracted from peripheral blood leukocytes using a GENOMIX Genome Extraction Kit (Biologica, Aichi, Japan) and quantified using a Qubit dsDNA BR Assay Kit (Thermo Fisher Scientific, MA, USA), and the quality of the DNA was assessed with a Tape Station (Agilent Technologies, CA, USA). Library preparation was carried out according to the manufacturer's instructions for the hybridization-based SureSeq Pan-Myeloid Panel (Oxford Gene Technology, Oxford, UK). The gene panel consists of 70 key genes implicated in myeloid malignancies (Table S1). Genomic DNA (500 ng, measured using Qubit) was fragmented to 187–205 bp using sonication (Covaris, MA, USA). The fragmented dsDNA was repaired with an ER enzyme mix to create blunt ends. PCR was subsequently performed to amplify the library before hybridization and target capture. The amplified library was denatured and captured with biotinylated probes. The hybridized gene targets were then bound to streptavidin beads and washed to remove any possible off-target DNA. After the capture of targets, PCR was used to add indexes that would identify the sample of each sequence in the next-generation sequencing (NGS) run. The dsDNA PCR products then included both index sequences and adaptor sequences. NGS was performed on a MiSeq system (Illumina, CA, USA) with 2 × 151 cycles using Reagent Kit v2 (300 cycles). Read sequencing was aligned to the human reference genome (GRCh37/hg19). Data were analyzed using Interpret NGS Analysis Software 3.5.40 (Oxford Gene Technology), which identified single-nucleotide variants (SNVs) and insertions or deletions (indels). Finally, low-frequency SNVs and indels suspected of being false positives were systematically inspected with IGV version 2.16.2. In addition, microarray analysis was performed using the Infinium OmniExpress Exome-8 v1.6 platform (Illumina) following the manufacturer's protocol. The analysis using GenomeStudio 2.0.3 (Illumina) included an analysis of both copy number and loss of heterozygosity events, with data visualization of the LogR ratio and B allele frequency (BAF) plots.

### Statistical analysis

The rate of CRc (CR + CRi; assessed according to the Revised International Working Group Criteria for AML) at the end of the induction cycles was calculated [21]. OS was calculated from the start of treatment to the date of death due to any cause. Event-free survival (EFS) was defined as the number of days from the start of treatment to persistent disease, relapse from CRc, or death, and relapse-free survival (RFS) was defined as the number of days from the first response (CRc) to relapse or death. The Clopper–Pearson interval was used to calculate the CIs, and the distribution of time-to-event endpoints, such as the median OS and its 90% CI, was estimated using the Kaplan–Meier method. Univariate logistic regression analysis, Cox proportional hazard regression analysis, and univariate Cox analysis were performed to estimate the 90% CIs, the *P* value, and the odds ratio or HR. The study assessed the association between individual gene mutations and the clinical outcomes of patients, applying the Benjamini–Hochberg multiple-testing correction.

## Results

### Patient characteristics

The baseline characteristics of the 29 patients who underwent genetic analysis are summarized in Table 1. There were 20 males and 9 females, and the median age was 68 years. Therapy-related AML was diagnosed in 2 patients (6.9%), AML with a history of MDS in 24 patients (82.8%), and de novo AML with karyotypic abnormalities characteristic of MDS in 3 patients (10.3%). According to the NCCN guidelines for AML, version 1.2019, 13 patients (44.8%) had an intermediate cytogenetic risk, and 16 patients (55.2%) had a poor cytogenetic risk. Twelve patients (41.4%) had complex karyotypes. The patient characteristics were similar to those of the entire NS87-P1-2 study [17].

**Table 1:** Baseline patient characteristics (N = 29)

Sex n (%)	
Male	20 (69.0)
Female	9 (31.0)
Age, years	
Mean (SD)	67.3 (4.2)
Median	68.0
Min, max	60.0, 74.0
ECOG PS n (%)	
0	14 (48.3)
1	13 (44.8)
2	2 (6.9)
Type of AML n (%)	
Therapy-related AML	2 (6.9)
AML with antecedent MDS	24 (82.8)
De novo AML with MDS karyotype	3 (10.3)
AML with antecedent CMML	0 (0.0)
Cytogenetic risk group n (%)	
Intermediate	13 (44.8)
Poor	16 (55.2)
White blood cell count × 10^3^/μL	
Mean (SD)	7.7 (13.2)
Median	2.2
Min, max	0.7, 54.9
Peripheral blasts %	
Mean (SD)	7.4 (9.1)
Median	3.0
Min, max	0, 29.0
Bone marrow blasts %	
Mean (SD)	36.0 (18.0)
Median	28.8
Min, max	20.0, 78.4

*SD* standard deviation, *ECOG* Eastern Cooperative Oncology Group, *PS* performance status, *AML* acute myeloid leukemia, *MDS* myelodysplastic syndromes, *CMML* chronic myelomonocytic leukemia

### Genetic analysis

**Fig. 1:**
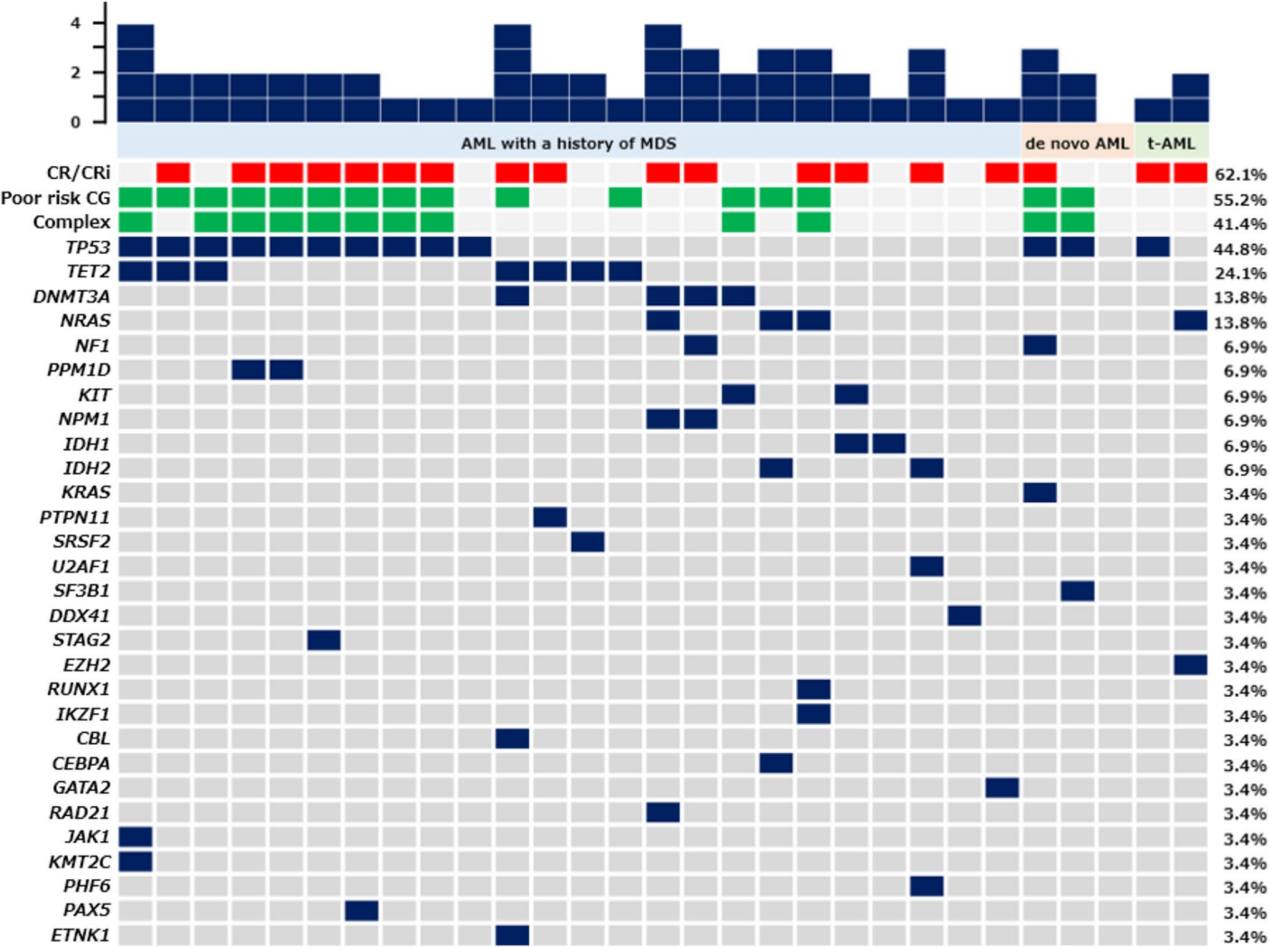
Genetic landscape of 29 patients with high-risk AML. The presence of CR/CRi (red), poor risk cytogenetics (CG) (green), complex (complex karyotypes) (green), and indicated mutations (navy blue) are displayed for each patient. The number of mutations in each patient is shown in the chart at the top. The frequency (%) of the mutations is shown to the right of the plot. *t-AML* therapy-related AML

At least one mutation was detected in 28 (96.6%) of the 29 patients assessed with NGS. Of these 28 patients, 8 (28.6%) had mutations in a single gene, 12 (42.9%) in two genes, 5 (17.9%) in three genes, and 3 (10.7%) in four genes. The most frequently mutated gene overall was *TP53* (n = 13, 44.8%). The genes with mutations observed in two or more patients were *TET2* (n = 7, 24.1%), *DNMT3A* and *NRAS* (n = 4 each, 13.8%), and *NF1*, *PPM1D*, *KIT*, *NPM1*, *IDH1*, and *IDH2* (n = 2 each, 6.9%) (Fig. 1). Of patients with *TP53* mutations (n = 13), 10 (76.9%) had complex karyotypes, 1 (7.7%) had a karyotype associated with del(7q) but did not have a complex karyotype, and the remaining 2 (15.4%) had normal karyotypes. In the current study, 5 (17.2%) had mutations specific for AML developed from MDS, in genes such as *SRSF2*, *SF3B1*, *U2AF1*, *ZRSR2*, *ASXL1*, *EZH2*, *BCOR*, or *STAG2*. When limited to AML with a history of MDS, one patient had an *SRSF2* mutation, one had a *U2AF1* mutation, and one had a *STAG2* mutation.

### Association between gene mutations and clinical outcomes

Of the 29 patients, 18 achieved CRc after one or two cycles of induction chemotherapy with NS-87/CPX-351. The CRc rate was 62.1% (90% CI 45.1–77.1) (Table 2). In the entire NS87-P1-2 study cohort, the CRc rate was 60.0% (90% CI 44.7–74.0; 21 out of 35 patients), and this rate is consistent with that in patients who underwent genetic analysis [17]. At the final analysis on October 26, 2023, the median OS of the 29 patients was 10.26 months (90% CI 4.87–18.18). The median duration of follow-up was 232 days (range 18–1292 days). The median EFS and RFS of these 29 patients were 3.48 months (90% CI 2.43–6.05) and 4.49 months (90% CI 2.20–5.46), respectively.

**Table 2:** Outcomes for patients with the most frequently occurring mutations

	All patients	*TP53*		*TET2*		*DNMT3A*		*NRAS*	
	(N = 29)	mut (N = 13)	wt (N = 16)	mut (N = 7)	wt (N = 22)	mut (N = 4)	wt (N = 25)	mut (N = 4)	wt (N = 25)
CR n (%)	14 (48.3%)	9 (69.2%)	5 (31.3%)	1 (14.3%)	13 (59.1%)	2 (50.0%)	12 (48.0%)	2 (50.0%)	12 (48.0%)
Odds ratio (90% CI)	–	4.95 (1.31, 18.68) *P* = 0.128		0.12 (0.02, 0.78) *P* = 0.128		1.08 (0.18, 6.37) *P* = 0.941		1.08 (0.18, 6.37) *P* = 0.941	
CR + CRi, n (%)	18 (62.1%)	9 (69.2%)	9 (56.3%)	3 (42.9%)	15 (68.2%)	3 (75.0%)	15 (60.0%)	3 (75.0%)	15 (60.0%)
Odds ratio (90% CI)	–	1.75 (0.48, 6.36) *P* = 0.571		0.35 (0.08, 1.51) *P* = 0.571		2.00 (0.27, 14.99) *P* = 0.571		2.00 (0.27, 14.99) *P* = 0.571	
Median OS (mos) (90% CI)	10.26 (4.87, 18.18)	7.43 (3.16, 14.96)	18.18 (5.33, 27.55)	3.48 (0.59, 24.46)	13.28 (6.28, 21.86)	6.28 (5.33, NA.)	10.26 (4.54, 18.18)	22.87 (13.28, NA.)	7.43 (4.54, 14.96)
Hazard ratio (90% CI)	–	2.55 (1.16, 5.62) *P* = 0.108		2.61 (1.15, 5.94) *P* = 0.108		0.56 (0.17, 1.92) *P* = 0.443		0.45 (0.16, 1.27) *P* = 0.275	
Median EFS (mos) (90% CI)	3.48 (2.43, 6.05)	2.43 (1.84, 3.81)	6.28 (3.48, 12.46)	1.84 (0.53, 3.48)	4.90 (2.43, 6.74)	6.28 (5.33, 14.27)	2.63 (2.20, 4.47)	9.75 (0.95, 14.27)	3.48 (2.43, 5.33)
Hazard ratio (90% CI)	–	3.65 (1.69, 7.87) ***P* = 0.012**		2.61 (1.13, 6.01) *P* = 0.116		0.51 (0.18, 1.42) *P* = 0.373		0.63 (0.25, 1.58) *P* = 0.407	
Median RFS (mos) (90% CI)	4.49 (2.20, 5.46)	1.48 (1.25, 2.86)	10.19 (5.06, 12.13)	2.2 (0.39, NA.)	5.06 (1.48, 10.19)	8.6 (5.06, 12.13)	2.86 (1.28, 5.46)	11.24 (5.29, 12.13)	2.86 (1.28, 5.46)
Hazard ratio (90% CI)	–	7.93 (2.53, 24.83) ***P* = 0.012**		2.05 (0.54, 7.73) *P* = 0.373		0.47 (0.13, 1.67) *P* = 0.373		0.50 (0.17, 1.52) *P* = 0.373	
Rates of HSCT n (%)	11 (37.9%)	6 (46.2%)	5 (31.3%)	1 (14.3%)	10 (45.5%)	2 (50.0%)	9 (36.0%)	2 (50.0%)	9 (36.0%)
Odds ratio (90% CI)	–	1.89 (0.53, 6.75) *P* = 0.595		0.20 (0.03, 1.35) *P* = 0.595		1.78 (0.30, 10.56) *P* = 0.595		1.78 (0.30, 10.56) *P* = 0.595	
1-year OS (%) (90% CI)	46.94 (30.93, 61.43)	30.77 (12.17, 51.71)	61.88 (38.73, 78.43)	28.57 (6.42, 56.46)	53.85 (34.89, 69.49)	37.50 (2.93, 76.15)	47.38 (30.29, 62.68)	100.00 (100.00, 100.00)	37.91 (21.84, 53.87)
2-year OS (%) (90% CI)	28.17 (14.38, 43.70)	0.00 (NA., NA.)	46.41 (24.33, 65.90)	28.57 (6.42, 56.46)	29.91 (13.97, 47.72)	37.50 (2.93, 76.15)	26.32 (12.29, 42.76)	50.00 (10.33, 80.93)	25.27 (11.10, 42.29)

The Benjamini–Hochberg procedure was applied to multiple hypothesis testing for *P* values. Statistically significant *P* values were indicated in bold font

*mut* mutated status, *wt* wild type, *CR* complete remission, *CI* confidence interval, *CRi* complete remission with incomplete blood count recovery, *OS* overall survival, *EFS* event-free survival, *RF*S relapse-free survival, *HSCT* hematopoietic stem cell transplantation, *NA* Not applicable

We assessed the association between individual gene mutations and patients’ clinical outcomes, with a focus on four genes, *TP53*, *TET2*, *DNMT3A*, and *NRAS*, that were mutated in at least four of the 29 patients (≥ 13.8%) (Table 2). *TP53* mutations belong to the adverse-risk factor in the European LeukemiaNet (ELN) 2022. Nevertheless, they were not associated with a decreased CRc rate in this study. The CRc rate was found to be similarly high in both patients with and without *TP53* mutations (69.2% vs. 56.3%; *P* = 0.571). However, patients with *TP53* mutations had a shorter OS, EFS, and RFS compared to patients with wild-type *TP53*. OS in patients with *TP53* mutations tended to be shorter than that in those with wild-type *TP53* (median OS: 7.43 months vs. 18.18 months; HR = 2.55 (90% CI 1.16–5.62), *P* = 0.108), and there were significant differences in EFS (median EFS: 2.43 months vs. 6.28 months; HR = 3.65 (90% CI 1.69–7.87), *P* = 0.012) and RFS (median RFS: 1.48 months vs. 10.19 months; HR = 7.93 (90% CI 2.53–24.83), *P* = 0.012).

In patients with *TET2* mutations, the CRc rate was comparable to that in patients with wild-type *TET2* (42.9% vs. 68.2%; *P* = 0.571). Moreover, patients with *TET2* mutations tended to have a shorter median OS compared to patients with wild-type *TET2* (median OS: 3.48 months vs. 13.28 months; HR = 2.61 (90% CI 1.15–5.94), *P* = 0.108). In patients with *DNMT3A* mutations, there were no significant differences in CRc rates or median OS compared to patients without *DNMT3A* mutations (CRc rate: 75.0% vs. 60.0%; *P* = 0.571, median OS: 6.28 months vs. 10.26 months; HR = 0.56 (90% CI 0.17–1.92), *P* = 0.443). Similarly, in patients with *NRAS* mutations, there were no significant differences in CRc rates or median OS, although patients with *NRAS* mutations tended to have a longer OS compared to patients with wild-type *NRAS* (CRc rate: 75.0% vs. 60.0%; *P* = 0.571, median OS: 22.87 months vs. 7.43 months; HR = 0.45 (90% CI 0.16–1.27), *P* = 0.275). These results indicate that CRc was achieved in patients who received NS-87/CPX-351 regardless of the mutation status of the *TP53*, *TET2*, *DNMT3A*, and *NRAS* genes. However, patients with *TP53* or *TET2* mutations showed a trend toward shorter OS compared to those with wild-type genes. Reaching definitive conclusions is difficult due to the small number of patients analyzed for these gene mutations.

In addition, differences in the CRc rate and the median OS of patients with 0 or 1 (n = 9), 2 (n = 12), or ≥ 3 (n = 8) gene mutations were not significant (CRc rate 44.4% vs. 66.7% vs. 75.0%; *P* = 0.408, median OS: 10.26 months vs. 6.79 months vs. 13.28 months; *P* = 0.528) (Table S2, S3). In addition, the CRc rate in patients with a poor cytogenetic risk was similar to the rate in patients with an intermediate cytogenetic risk (62.5% vs. 61.5%; *P* = 0.958). The OS of patients with a poor cytogenetic risk was shorter compared to those with an intermediate risk, but the difference was not significant (8.25 months vs. 10.26 months; *P* = 0.179). NS-87/CPX-351 demonstrated a certain level of efficacy even in patients with adverse-risk cytogenetics.

## Discussion

Compared to primary AML, secondary AML, including AML evolved from MDS and therapy-related AML, is generally associated with a poorer prognosis [5, 6]. NS-87/CPX-351 showed efficacy equivalent to that of conventional 7 + 3 chemotherapy in a phase 2 study of newly diagnosed AML patients, but it showed superior efficacy compared to 7 + 3 chemotherapy when given to patients with high-risk AML, which consists of therapy-related AML or AML-MRC [22]. However, the reason why NS-87/CPX-351 is more effective in these subtypes remains elusive. The current study investigated the relationship between genetic characteristics and treatment outcomes for NS-87/CPX-351 in elderly Japanese patients with therapy-related AML or AML-MRC, for whom genetic analysis was available in the NS87-P1-2 study, in order to explore gene mutations that predict the effectiveness of NS-87/CPX-351.

A high proportion of mutations in *TP53* (44.8%), *TET2* (24.1%), *DNMT3A* (13.8%), and *NRAS* (13.8%) were noted in this study of elderly Japanese patients with high-risk AML. Gene mutations reported in the genetic analysis of the 301 study indicated that ≥ 20% of patients overall had mutations in *TP53* (34.9%), *ASXL1* (29.6%), *RUNX1* (25.4%), *TET2* (25.4%), and *DNMT3A* (24.3%) [18]. The eligibility criteria for the NS87-P1-2 study were the same as those for the 301 study, and *TP53* mutations were typically found to be the most frequent among this high-risk AML population. *TP53* mutations are present in approximately 5–8% of all AML patients and are associated with a poor prognosis regardless of treatment regimen [23]. In the current study, 45% of the patients had *TP53* mutations, but the subjects of this study were patients with AML-MRC and therapy-related AML, so a high proportion of *TP53* mutations is not unexpected. The current study also noted a high frequency of mutations in the *TET2* and *DNMT3A* genes, as in the 301 study. Mutations in genes encoding epigenetic modifiers, such as *TET2* and *DNMT3A*, are more common in elderly patients and are often present in the founding clone [23]. Thus, since patients aged 60–75 were eligible for the current study, this result is reasonable. In addition, *NRAS* mutations, which are associated with signaling pathways and a poor prognosis, are frequently observed in secondary AML [8].The gene mutations frequently observed in the current study are considered to reflect the profile of elderly high-risk AML patients.

In the genetic analysis of the 301 study and the French cohort study, NS-87/CPX-351 was found to offer little benefit to AML patients with *TP53* mutations. In the 301 study, the CRc rate in patients with *TP53* mutations was 29% (7/24) compared to 48% in the entire study population [16, 18]. In addition, in the French cohort, the CRc rate in patients with *TP53* mutations was significantly lower than that in patients with wild-type *TP53* (41% (9/22) vs. 66% (35/53); *P* = 0.04) [24]. In the current study, the rate in patients with *TP53* mutations was 69% (9/13), which is high and comparable to that in patients with wild-type *TP53*. This contrasts with the 301 study and the French cohort study. However, in the Italian study, the CRc rates in patients with and without *TP53* mutations were 77% (10/13) and 75% (18/24) (*P* = 1.0), respectively [19], and in the German study, 54% and 47% (*P* = 0.77), respectively [20]. The results of the CRc rate in patients with *TP53* mutations in the Japanese cohort are similar to those reported in the retrospective studies of the Italian and German cohorts. However, the current study confirmed that patients with *TP53* mutations have a poorer prognosis compared to patients with wild-type *TP53*, with significantly shorter EFS and RFS.

In the genetic analysis of the 301 study, the median OS was longer in the NS-87/CPX-351 group compared to the 7 + 3 chemotherapy group among patients with *DNMT3A* mutations (12.6 months vs. 5.5 months; HR = 0.41 (95% CI 0.19–0.89)) and *TET2* mutations (9.1 months vs. 3.7 months; HR = 0.47 (95% CI 0.23–0.93)) [18]. In the current study, the presence of *DNMT3A* and *TET2* mutations did not have a favorable impact on the OS in Japanese AML patients. A point worth noting is that the current study compared differences in outcomes for NS-87/CPX-351 between patients with and without mutations, while the 301 study compared clinical outcomes of patients with mutations who received NS-87/CPX-351 and those who received 7 + 3 chemotherapy. In the current study, there were no significant differences in efficacy between patients with mutated and wild-type *NRAS*, as a certain level of efficacy was displayed in both groups. Recent studies have reported that patients treated with NS-87/CPX-351 who have a co-mutation in the *RAS* pathway (*NRAS*/*KRAS*) experienced poor prognosis [25]. In the current study, however, none of the patients with *NRAS* mutations (n = 4) also had co-mutations with *KRAS*. There were only a small number of patients with mutations in *TET2* (n = 7), *DNMT3A* (n = 4), and *NRAS* (n = 4), so further confirmation is required in a larger patient population.

Overall, these findings indicate that the CRc rate demonstrated by NS-87/CPX-351 was not influenced by gene mutations and that gene mutations alone failed to identify responsive patients. However, patients with *TP53* mutations often experience relapse or disease progression after achieving remission, suggesting that this treatment may not contribute to prolonged OS. In contrast, there was a high response rate to NS-87/CPX-351, even in patients with *TP53* mutations. In patients with high-risk AML for which NS-87/CPX-351 is approved, achieving remission is extremely important as allogeneic hematopoietic stem cell transplantation is recommended with the first complete remission [26]. Of the 11 patients who underwent transplantation after receiving NS-87/CPX-351 in the current study (Table S4), as many as 9 patients did so not in remission but in relapse or disease progression, so there was no significant difference in median OS between transplant patients and non-transplant patients (median OS: 13.28 months vs. 5.57 months; *P* = 0.456) (Table S5). Similarly, in patients with *TP53* mutations, there was no significant difference in median OS, although transplant patients tended to have a longer OS compared to non-transplant patients (median OS: 9.25 months vs. 3.16 months; HR = 0.43 (90% CI 0.15–1.27), *P* = 0.199) (Table S5). Of the two patients with wild-type *TP53* who underwent transplantation in CRc, one patient was lost to follow-up due to transfer for transplantation, while the other patient survived for over 2 years until the study completion date. In the 301 study, most patients (40 out of 53) underwent transplantation during CRc, resulting in prolonged OS after transplantation [27]. The observation of shorter RFS in Japanese patients with *TP53* mutations suggests the importance of undergoing transplantation soon after achieving the first complete remission following NS-87/CPX-351 treatment.

In conclusion, this study found no gene mutations directly associated with the efficacy of NS-87/CPX-351 in elderly Japanese patients with high-risk AML. While NS-87/CPX-351 achieved remission even in patients with *TP53* mutations, these patients demonstrated a higher risk of relapse. Therefore, our findings suggest that there is a need to consider such treatment strategies as early transplantation following the achievement of remission with NS-87/CPX-351 treatment, especially in patients with *TP53* mutations.

## Limitations

This study involved a small sample of patients who met the criteria for the NS87-P1-2 study and who provided informed consent for genetic analysis, and not all the patients included in the NS87-P1-2 study had samples available for NGS analysis. Further studies need to be performed to identify factors predicting treatment outcomes for NS-87/CPX-351.

## Supplementary Information

Below is the link to the electronic supplementary material.

Supplementary file1 (DOCX 52 KB)

## Data Availability

All data generated or analyzed during this study are included in this published article and its supplementary information files.
