# Supplementary material for: Association between gene mutations and outcomes in Japanese high-risk AML patients: a phase 1/2 study of NS-87/CPX-351
Source: Int J Hematol. 2025 Feb 27;122(1):57–65. doi: 10.1007/s12185-025-03956-8 (PMC12202506; doi:10.1007/s12185-025-03956-8)
Supplement: Supplementary file 1 — Supplementary file1 (DOCX 52 KB) [file 12185_2025_3956_MOESM1_ESM.docx]

**Supplementary Material**

**Inclusion and exclusion criteria for the NS87-P1-2 study**

[Inclusion criteria]

Patients who met all of the following criteria and provided written informed consent

1. Japanese patients aged 60–75 years at the time of signing the informed consent form
2. Patients diagnosed with AML according to the WHO criteria published in 2017
3. Patients with AML corresponding to one of the following

- Therapy-related AML

Requires prior cytotoxic therapy for an unrelated disease:

・alkylating agents

・ionizing radiation therapy: large fields including active bone marrow

・topoisomerase II inhibitors

・other: antimetabolites, antitubulin agents

- AML with a history of MDS

Requires any of the following:

・Previous confirmed history of MDS

・Bone marrow evidence of dysplasia present in ≥10% of cells in one or more myeloid lineages or ≥10% dysplastic megakaryotypes

・Unequivocal dysplasia in <10% of cells in one or more myeloid cell lines with clonal abnormalities characteristic of MDS (one of the following)

Clonal abnormalities: Unbalanced changes: +8*, -7 or del(7q), -5 or del(5q); del(20q)*, -Y*, i(17q) or t(17p), -13 or del(13q), del(11q), del(12p) or t(12p), del(9q), idic(X)(q13) Balanced changes: t(11;16)(q23;p13.3); t(3;21)(q26.2;q22.1); t(1;3)(p36.3;q21.2); t(2;11)(p21;q23); inv(3)(q21q26.2), t(6:9)(p23;q34)

*If the sole cytogenetic abnormality, also requires morphologic criteria with dysplasia present in ≥10% of cells in one or more myeloid lineages or ≥10% dysplastic megakaryotypes; all other clonal abnormalities are sufficient for a presumptive diagnosis.

- *De novo* AML with karyotypic abnormalities characteristic of MDS

Requires any of the following:

・Complex karyotype (defined as 3 or more chromosomal abnormalities)

・Unbalanced: -7 or del(7q); -5 or del(5q); i(17q) or t(17p); -13 or del(13q); del(11q); del(12p) or t(12p); del(9q); idic(X)(q13)

・Balanced: t(11;16)(q23;p13.3); t(3;21)(q26.2;q22.1); t(1;3)(p36.3;q21.2); t(2;11)(p21;q23), t(5;12)(q33;p12); t(5;7)(q33;q11.2); t(5;17)(q33;p13); t(5;10)(q33;q21); t(3;5)(q25;q34)

- AML with a history of CMML

Requires a history of CMML, which in turn requires the following (at diagnosis of CMML):

・Peripheral blood monocytosis >1000/μL

・Absence of Philadelphia chromosome or BCR-ABL1 fusion gene

・In the presence of eosinophilia, absence of rearrangements of PDGFRA or PDGFRB

・Presence of dysplasia in one or more myeloid lineages

・If myelodysplasia is absent/minimal, CMML may still be diagnosed if the above requirements are met and there is either:

・the presence of an acquired clonal cytogenetic or molecular genetic abnormality in hematopoietic cells or

・the persistence of monocytosis for ≥ 3 months and all other causes of monocytosis have been excluded

・There are fewer than 20% blasts (myeloblasts, monoblasts, or promonocytes) in the peripheral blood and bone marrow

1. Eastern Cooperative Oncology Group (ECOG) performance status of 0, 1, or 2
2. Patients whose laboratory results meet the following criteria within 3 days before the start of the study drug:

- Serum creatinine < 2.0 mg/dL
- Serum bilirubin < 2.0 mg/dL (patients with Gilbert's syndrome should be judged by a specialist)
- Serum aspartate aminotransferase (AST) and alanine aminotransferase (ALT) < 3.0 times the upper limit of normal (ULN) (patients whose liver enzymes exceed ULN in relation to AML should be judged by a specialist)

1. Patients whose cardiac ejection fraction ≥ 50% according to echocardiography or a multiple-gated acquisition (MUGA) scan within 28 days prior to the start of the study drug
2. Patients who have agreed to use appropriate contraception (for example, contraceptive devices or contraceptive drugs) during the treatment period and for 6 months after administration
3. Patients providing written informed consent
4. Patients who were deemed by a physician to be able to survive for more than 12 weeks after the start of treatment

[Exclusion criteria]

Patients who met any of the following criteria were excluded from the study.

- 1. Patients with a history of MPN (defined as a history of essential thrombocytosis or polycythemia vera, or idiopathic myelofibrosis except for CMML) or combined MDS/MPN prior to the diagnosis of AML
  2. Patients with acute promyelocytic leukemia [t(15;17)] or favorable cytogenetics, including t(8;21) or inv16
  3. Patients with clinical evidence of active CNS leukemia
  4. Patients with active (uncontrolled, metastatic) second malignancies

Patients who have remained in remission according to imaging or tumor marker tests without recurrence for at least 6 months after completion of cytotoxic therapy can be enrolled (maintenance therapy with hormonal agents after remission, etc., is not counted as cytotoxic therapy).

- 1. Patients who previously received induction therapy for AML

Hydroxyurea for disease control can be permitted until 24 hours before the start of the study treatment. Patients previously treated with a hypomethylating agent (HMA), cytarabine (AraC) alone (>1 g/m^2^/day), AraC plus an anthracycline or HSCT, etc. for AML are also excluded.

- 1. Patients who received any therapy for MDS within two weeks of the first dose of the study drug

Adverse reactions associated with prior MDS treatment must have recovered to Grade 1 or less based on CTCAE v5.0 prior to start of the study treatment.

- 1. Patients who underwent any surgery (excluding local surgery) or radiation therapy within four weeks of the first dose of the study drug
  2. Patients with prior cumulative anthracycline exposure of greater than 368 mg/m^2^ (218 mg/m^2^ for patients who received radiation therapy to the mediastinum) daunorubicin or the equivalent
  3. Patients with any serious medical condition, laboratory abnormality, or psychiatric illness that would prevent the obtaining of informed consent
  4. Patients with myocardial impairment due to any cause (e.g., cardiomyopathy, ischemic heart disease, significant valvular dysfunction, hypertensive heart disease, and congestive heart failure) resulting in heart failure according to the New York Heart Association Criteria (Class III or IV staging)
  5. Patients with an active or uncontrolled infection

Patients with an infection for which they are receiving treatment (antibiotics, antifungals, or antivirals) can be enrolled but must be afebrile and hemodynamically stable for more than 72 hours.

- 1. Patients with current evidence of an invasive fungal infection (patients with a suspected infection must have a subsequent negative culture to be eligible) or known HIV
  2. Patients with current evidence of an active hepatitis B or C infection (with rising transaminase values)
  3. Patients with hypersensitivity to cytarabine, daunorubicin or liposomal products
  4. Patients with a history of Wilson’s disease or other copper metabolism disorder
  5. Patients who received any treatment with another investigational drug or unapproved drug within 28 days of the first dose of the study drug
  6. Pregnant women, women suspected of being pregnant, and lactating women

Female patients should undergo a pregnancy test during the screening phase. However, this test is not required for menopausal patients who have been in menopause for more than one year since their last period without any other medical reason or for female patients who cannot become pregnant due to a hysterectomy or ovary removal. Interruption of breastfeeding will not allow inclusion in this study.

- 1. Patients who were deemed to be inappropriate for the study by the investigator (subinvestigator)

# **Tables**

**Table S1 List of target genes and their exons**

| **Gene** | **Exons** | **Gene** | **Exons** | **Gene** | **Exons** | **Gene** | **Exons** | **Gene** | **Exons** |
| --- | --- | --- | --- | --- | --- | --- | --- | --- | --- |
| ***ABL1*** | **All** | ***CTNNA1*** | **All** | ***GNB1*** | **5-6** | ***MYD88*** | **3,5** | ***RUNX1*** | **All** |
| ***ASXL1*** | **12** | ***CUX1*** | **All** | ***HRAS*** | **All** | ***NF1*** | **All** | ***SETBP1*** | **4** |
| ***BCOR*** | **All** | ***CXCR4*** | **All** | ***IDH1*** | **4** | ***NFE2*** | **All** | ***SF3B1*** | **13-16** |
| ***BCORL1*** | **All** | ***DDX41*** | **All** | ***IDH2*** | **4,5** | ***NOTCH1*** | **26-28, 34,3’UTR** | ***SH2B3*** | **All** |
| ***BIRC3*** | **All** | ***DNMT3A*** | **All** | ***IKZF1*** | **All** | ***NPM1*** | **12** | ***SRSF2*** | **1** |
| ***BRAF*** | **15** | ***EP300*** | **All** | ***IRF1*** | **All** | ***NRAS*** | **2,3** | ***STAG2*** | **All** |
| ***BTK*** | **15** | ***ETNK1*** | **All** | ***JAK1*** | **All** | ***PAX5*** | **All** | ***STAT3*** | **19-21** |
| ***CALR*** | **9** | ***ETV6*** | **All** | ***JAK2*** | **12,14** | ***PDGFRA*** | **All** | ***STAT5B*** | **16** |
| ***CBL*** | **8,9** | ***EZH2*** | **All** | ***JAK3*** | **All** | ***PHF6*** | **All** | ***TET2*** | **3-11** |
| ***CDKN2A*** | **All** | ***FBXW7*** | **9-11** | ***KIT*** | **2,8-11, 13,17** | ***PLCG2*** | **19,20,24** | ***TP53*** | **All** |
| ***CEBPA*** | **All** | ***FLT3*** | **13-15,20** | ***KMT2A*** | **All** | ***PPM1D*** | **All** | ***U2AF1*** | **2,6** |
| ***CHEK2*** | **All** | ***GATA1*** | **All** | ***KMT2C*** | **All** | ***PTEN*** | **All** | ***WT1*** | **7,9** |
| ***CREBBP*** | **All** | ***GATA2*** | **2-6** | ***KRAS*** | **2,3** | ***PTPN11*** | **3,13** | ***XPO1*** | **All** |
| ***CSF3R*** | **14-17** | ***GNAS*** | **8-10** | ***MPL*** | **10** | ***RAD21*** | **All** | ***ZRSR2*** | **All** |

**Table S2 Summary of univariate logistic regression of the response (CR or CRi) rate**

| Factor | Factor level | CR+CRi n (%) | Odds ratio  (90% CI) | Pairwise  *P* value | Overall  *P* value |
| --- | --- | --- | --- | --- | --- |
| All patients | - (N=29) | 18 (62.1) | - | - | - |
| Age | 60-69 (N=20) | 13 (65.0) | 1.49  (0.39, 5.71) | 0.629 | 0.629 |
|  | 70-75 (N=9) | 5 (55.6) |  |  |  |
| ECOG PS | 0 (N=14) | 7 (50.0) | NA.  (NA., NA.) | 0.979 | 0.601 |
|  | 1 (N=13) | 9 (69.2) | NA.  (NA., NA.) | 0.983 |  |
|  | 2 (N=2) | 2 (100.0) |  |  |  |
| Cytogenetic risk group | Intermediate (N=13) | 8 (61.5) | 0.96  (0.27, 3.40) | 0.958 | 0.958 |
|  | Poor (N=16) | 10 (62.5) |  |  |  |
| WBC category (10^3^/μL) | < 20 (N=25) | 14 (56.0) | NA.  (NA., NA.) | 0.959 | 0.959 |
|  | >= 20 (N=4) | 4 (100.0) |  |  |  |
| Peripheral blasts count (%) | < 10 (N=20) | 12 (60.0) | 0.75  (0.19, 2.99) | 0.732 | 0.732 |
|  | >= 10 (N=9) | 6 (66.7) |  |  |  |
| Bone marrow blast count (%) | 20-40 (N=21) | 13 (61.9) | NA.  (NA., NA.) | 0.977 | 0.441 |
|  | >40-60 (N=4) | 1 (25.0) | NA.  (NA., NA.) | 0.967 |  |
|  | >60 (N=4) | 4 (100.0) |  |  |  |
| Number of mutations | 0-1 (N=9) | 4 (44.4) | 0.27  (0.05, 1.52) | 0.184 | 0.408 |
|  | 2 (N=12) | 8 (66.7) | 0.67  (0.12, 3.57) | 0.752 |  |
|  | >=3 (N=8) | 6 (75.0) |  |  |  |

CR: complete remission; CRi: complete remission with incomplete blood count recovery; CI: confidence interval; ECOG PS: Eastern Cooperative Oncology Group performance status; N: number of patients with available data for the specified factor; n: number of patients with CR or CRi described by the specified factor; *P* value: two-sided *P* value from a Wald chi-square test; Pairwise *P* value: *P* values for levels within each factor; Overall *P* value: *P* values for each factor; NA: not applicable.

**Table S3 Summary of univariate Cox proportional hazard regression of OS**

| Factor | Factor level | Median OS (mos)  (90% CI) | Hazard ratio  (90% CI) | Pairwise  *P* value | Overall  *P* value |
| --- | --- | --- | --- | --- | --- |
| All patients | - (N=29) | 10.26  (4.87, 18.18) | - | - | - |
| Age | 60-69 (N=20) | 10.26  (5.33, 21.86) | 0.81  (0.37, 1.80) | 0.668 | 0.668 |
|  | 70-75 (N=9) | 4.87  (1.15, NA.) |  |  |  |
| ECOG PS | 0 (N=14) | 6.79  (2.47, 18.18) | NA.  (NA., NA.) | 0.993 | 0.816 |
|  | 1 (N=13) | 10.26  (4.54, 21.86) | NA.^)^  (NA., NA.) | 0.993 |  |
|  | 2 (N=2) | NA.  (NA., NA.) |  |  |  |
| Cytogenetic risk group | Intermediate (N=13) | 10.26  (4.54, 27.55) | 0.52  (0.24, 1.16) | 0.179 | 0.179 |
|  | Poor (N=16) | 8.25  (4.11, 18.18) |  |  |  |
| WBC category (10^3^/μL) | < 20 (N=25) | 10.26  (4.54, 18.18) | 1.90  (0.56, 6.47) | 0.386 | 0.386 |
|  | >= 20 (N=4) | NA.  (4.87, NA.) |  |  |  |
| Peripheral blast count  (%) | < 10 (N=20) | 7.84  (4.11, 14.96) | 1.92  (0.85, 4.34) | 0.188 | 0.188 |
|  | >= 10 (N=9) | 24.46  (3.16, NA.) |  |  |  |
| Bone marrow blast count (%) | 20-40 (N=21) | 7.43  (4.11, 14.96) | 10.04  (1.77, 57.13) | **0.029** | 0.078 |
|  | >40 - 60 (N=4) | 13.28  (6.28, 24.46) | 6.32  (0.89, 44.98) | 0.122 |  |
|  | >60 (N=4) | NA.  (2.47, NA.) |  |  |  |
| Number of mutations | 0-1 (N=9) | 10.26  (1.15, 25.48) | 1.32  (0.50, 3.47) | 0.640 | 0.528 |
|  | 2 (N=12) | 6.79  (3.48, 21.86) | 1.84  (0.74, 4.56) | 0.273 |  |
|  | >=3 (N=8) | 13.28  (3.16, NA.) |  |  |  |

OS: overall survival; CI: confidence interval; ECOG PS: Eastern Cooperative Oncology Group performance status; N: number of patients with available data for the specified factor; *P* value: two-sided *P* value from a Wald chi-square test; Pairwise *P* value: *P* values for levels within each factor; Overall *P* value: *P* values for each factor; NA: not applicable.

**Table S4 Baseline characteristics of transplant patients**

|  | | N=11 |
| --- | --- | --- |
| Sex n (%) | | |
|  | Male | 7 (63.6) |
|  | Female | 4 (36.4) |
| Age, years | | |
|  | Mean (SD) | 64.4 (3.7) |
|  | Median | 64.0 |
|  | min, max | 60.0, 69.0 |
| Time from diagnosis to HSCT, days | | |
|  | Mean (SD) | 147.5 (47.4) |
|  | Median | 143 |
|  | min, max | 83, 230 |
| Treatments received before HSCT n (%) | | |
|  | NS-87/CPX-351 | 11 (100.0) |
|  | 7 + 3 chemotherapy | 1 (9.1) |
|  | MEC | 1 (9.1) |
|  | Venetoclax + azacitidine | 2 (18.2) |
|  | CAG | 1 (9.1) |
|  | Azacitidine | 1 (9.1) |
| Graft type n (%) | | |
|  | Bone marrow | 1 (9.1) |
|  | Peripheral blood | 5 (45.5) |
|  | Cord blood | 5 (45.5) |
| Remission status before HSCT n (%) | | |
|  | CRc | 2 (18.2) |
|  | Non-CRc | 9 (81.8) |

SD: standard deviation; HSCT: hematopoietic stem cell transplantation; MEC: mitoxantrone, etoposide, and cytarabine; CAG: cytarabine, aclarubicin and G-CSF; CRc: composite complete remission.

|  | All patients  (N=29) | | *TP53* mut  (N=13) | |
| --- | --- | --- | --- | --- |
|  | HSCT  (N=11) | Non-HSCT  (N=18) | HSCT  (N=6) | Non-HSCT  (N=7) |
| Median OS (mo)  (90% CI) | 13.28  (7.43, 27.55) | 5.57  (3.16, 18.18) | 9.25  (4.54, NA.) | 3.16  (0.59, NA.) |
| Hazard ratio  (90% CI) | 0.72  (0.34, 1.49)  *P*=0.456 | | 0.43  (0.15, 1.27)  *P*=0.199 | |
| Post HSCT  Median OS (mo)  (90% CI) | 7.20  (1.78, 19.04) | NA. | 4.85  (1.02, NA.) | NA. |
| Relapse rate  n (%) | N=8  6 (75.0) | N=10  8 (80.0) | N=5  5 (100.0) | N=4  4 (100.0) |
| Median EFS (mo)  (90% CI) | 6.05  (3.81, 12.46) | 4.88  (2.43, 11.51) | 9.25  (4.54, NA.) | 3.16  (0.59, NA.) |
| Hazard ratio  (90% CI) | 0.79  (0.40, 1.58)  *P*=0.576 | | 0.84  (0.32, 2.20)  *P*=0.767 | |
| Median RFS (mo)  (90% CI) | 3.39  (1.48, 11.24) | 5.18  (1.25, 10.19) | 2.73  (1.28, NA.) | 1.25  (0.39, NA.) |
| Hazard ratio  (90% CI) | 0.80  (0.33, 1.94)  *P*=0.684 | | 0.81  (0.24, 2.80)  *P*=0.784 | |
| 1-year OS (%)  (90% CI) | 51.14  (23.95, 73.00) | 44.44  (25.06, 62.18) | 33.33  (7.37, 62.95) | 28.57  (6.42, 56.46) |
| 2-year OS (%)  (90% CI) | 25.57  (6.27, 51.10) | 29.63  (12.70, 48.82) | 0.00  (NA., NA.) | 0.00  (NA., NA.) |

**Table S5 Outcomes for transplant patients and non-transplant patients**

mut: mutated status; HSCT: hematopoietic stem cell transplantation; OS: overall survival; mo: month; CI: confidence interval; EFS: event-free survival; RFS: relapse-free survival; NA, not applicable.
